# Tarsal tunnel syndrome caused by a flexor digitorum accessorius longus muscle: a case report and review of the literature

**DOI:** 10.1186/1757-1146-4-S1-O42

**Published:** 2011-05-20

**Authors:** Dean Samaras

**Affiliations:** 1 Kingsford Podiatry Group, Melbourne, Victoria, 3000, Australia

## 

The flexor digitorum accessorius longus (FDAL) is a rare muscular occurrence in the lower extremity. In this case study and review of the literature the cause of tarsal tunnel syndrome by a flexor digitorum accessorius longus is described with emphasis on clinical testing methods, diagnostic imaging and both non-surgical and surgical management. A 52 year old male presented with pain and paraesthesia in his left foot and ankle. Tinel’s sign was exhibited clinically as well as severe bilateral pes planus. Inverted style custom foot orthoses failed to reduce the patient’s symptoms over a six month period. Magnetic resonance imaging (MRI) confirmed the presence of a FDAL muscle within the tarsal tunnel. The patient was subsequently referred to a podiatric surgeon for decompression and excision of the accessory muscle. Follow up at 18 months revealed resolution of pain and return to normal activities however, some plantar paraesthesia remained. A literature search was conducted across several major scientific databases. Further information was sought with citation tracking, reference checking, reviewing unpublished data and seeking expert opinion. The search yielded sixteen research papers pertaining to the FDAL muscle. Six of these papers described the FDAL as the cause of TTS in the form of case studies and were included in the review. Pain and paraesthesia were described as the pertinent symptoms and indicative of neural pathology, as was a Tinel’s sign. MRI was preferred for diagnostic purposes due to the ability to detect space-occupying lesions within the tarsal tunnel. In some cases FDAL was diagnosed intra-operatively. Surgical decompression via excision of the accessory muscle was described with a reportedly high success rate. Practitioners treating the foot and ankle should have a degree of suspicion for the presence of an accessory muscle particularly when interpreting MRI films as part of the diagnostic work-up for TTS.

